# Water-Soluble Alkali Lignin as a Natural Radical Scavenger and Anticancer Alternative

**DOI:** 10.3390/ijms241612705

**Published:** 2023-08-11

**Authors:** Donika Ivanova, Galina Nikolova, Yanka Karamalakova, Severina Semkova, Vania Marutsova, Zvezdelina Yaneva

**Affiliations:** 1 Department of Pharmacology, Animal Physiology, Biochemistry and Chemistry, Faculty of Veterinary Medicine, Trakia University, Student Campus, 6000 Stara Zagora, Bulgaria; zvezdelina.yaneva@trakia-uni.bg; 2 Department of Medical Chemistry and Biochemistry, Medical Faculty, Trakia University, 11 “Armeyska” St., 6000 Stara Zagora, Bulgaria; galina.nikolova@trakia-uni.bg (G.N.); yanka.karamalakova@trakia-uni.bg (Y.K.); 3 Department of Electroinduced and Adhesive Properties, Institute of Biophysics and Biomedical Engineering, Bulgarian Academy of Sciences, 1040 Sofia, Bulgaria; severina.yordanova@gmail.com; 4 Department of Internal Diseases, Faculty of Veterinary Medicine, Trakia University, Student Campus, 6000 Stara Zagora, Bulgaria; vaniamarutsova@abv.bg

**Keywords:** alkali lignin, antioxidant activity, UV-B irradiation, anti-proliferative activity, cancer treatment, biomaterial

## Abstract

Several phytochemicals, which display antioxidant activity and inhibit cancer cell phenotypes, could be used for cancer treatment and prevention. Lignin, as a part of plant biomass, is the second most abundant natural biopolymer worldwide, and represents approximately 30% of the total organic carbon content of the biosphere. Historically, lignin-based products have been viewed as waste materials of limited industrial usefulness, but modern technologies highlight the applicability of lignin in a variety of industrial branches, including biomedicine. The aims of our preliminary study were to compare the antioxidant properties of water-soluble alkali lignin solutions, before and after UV-B irradiation, as well as to clarify their effect on colon cancer cell viability (Colon 26), applied at low (tolerable) concentrations. The results showed a high antioxidant capacity of lignin solutions, compared to a water-soluble control antioxidant standard (Trolox) and remarkable radical scavenging activity was observed after their UV-B irradiation. Diminishment of cell viability as well as inhibition of the proliferative activity of the colon cancer cell line with an increase in alkali lignin concentrations were observed. Our results confirmed that, due to its biodegradable and biocompatible nature, lignin could be a potential agent for cancer therapy, especially in nanomedicine as a drug delivery system.

## 1. Introduction

In recent years, the administration of natural bioflavonoids, such as resveratrol [1,2], quercetin [3,4], curcumin [5,6], and (−)-epigallocatechin-3-gallate [7] etc., has demonstrated anti-carcinogenic activity and some of these natural bioflavonoids, in combination with conventional and new-generation anticancer drugs, also exhibited synergistic cytotoxic effect towards cancer cells [8,9,10,11,12,13]. Because of dose-limiting side effects as well as induced drug resistance, the development of novel therapeutic strategies in which natural products and anticancer chemotherapeutic drugs are in tight cooperation, is crucially necessary [8,9,11,14].

**Scheme 1:**
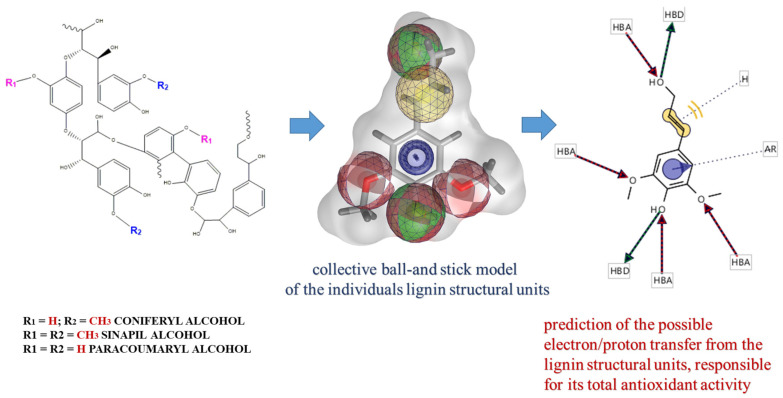
Representative molecular lignin structural units and possibilities of their involvement into processes of electron/proton exchange in aqueous solution.

Several phytochemicals, which display antioxidant activity and inhibit cancer cell phenotypes, could be used for cancer treatment and prevention. Some of these compounds (curcumin, epigallocatechin gallate, and paclitaxel etc.) have already been developed as Food-and-Drug-Administration (FDA, USA)-approved drugs for cancer therapy. Because they are abundant in food sources and have low levels of side effects, an increasing number have been developed as health supplements [15,16,17]. Lignin, as a part of plant biomass, is the second most abundant natural biopolymer worldwide, and represents approximately 30% of the total organic carbon content of the biosphere [18,19]. Chemically, it is a complex phenolic nonlinear biopolymer, which consists mainly of three monomer structural units, generally called monolignols (p-hydroxyphenyl, guaiacyl, and syringyl), for which the chemical formulas are indicated in Scheme 1 [20]. Their individual contribution into the lignin structure varies significantly, depending on the type of the extraction process [21,22]. Historically, lignin-based products have been viewed as waste materials of limited industrial usefulness, but modern technologies highlight the applicability of lignin in variety of industrial branches, including biomedicine [23]. Because of the diverse functional groups (carboxyl, hydroxyl, and sulfhydryl) present in their structure, lignin and its derivatives have been included in different pharmacological studies and have been reported as compounds with the potential to treat diseases, like diabetes, and emphysema [24,25]. There is also information about the antioxidant, antiviral, antimicrobial, anticoagulant, and immune modulator capabilities of lignin and its derivatives [26,27,28,29]. Due to their biodegradability and biocompatibility, lignins possess characteristic marks to be involved in nanomedicine as precursors for the development of drug-delivery system(s) [30,31], but additional studies on their effect on intracellular homeostasis have to be performed.

The aims of our preliminary study were to compare the antioxidant properties of water-soluble alkali lignin solutions, before and after UV-B irradiation, as well as to clarify the effect of alkali lignin solutions on colon cancer cell viability (Colon 26), applied at low (tolerable) concentrations.

## 2. Results and Discussion

### 2.1. Total Antioxidant Capability of Alkali Lignin Water Solutions

The total antioxidant ability of lignin water solutions (1 mg/mL and 3 mg/mL) were screened by the DPPH (2,2-diphenyl-1-picrylhydrazyl) and ABTS (2,2′-azino-bis(3-ethylbenzothiazoline-6-sulfonic acid) assay methods, using colorimetric spectrophotometric analyses and a hydrophilic analog of vitamin E, Trolox (6-hydroxy-2,5,7,8-tetramethylchroman-2-carboxylic acid), as a water-soluble control antioxidant standard. It has been shown that Trolox, applied at low concentrations, demonstrated significant antioxidant activity towards the endogenous ROS production of HeLa cells, compared to its high concentration, where prooxidant properties have been exhibited [32].

Both methods (DPPH and ABTS) are among the most popular assays for antioxidant activity determination [33]. The mechanisms are based on the transfer of a single electron/proton from the antioxidant compound(s) to stable radical forms, by which chemical reduction reactions have been initiated and the radical structures have been neutralized. In our experiments, we used alkali lignin solutions to eliminate DPPH and ABTS radicals, which is cross-linked with the potential of the heterobiopolymer to scavenge radical formations. The specific chemical structures of different lignin structural units as well as the specific functional groups involved in the transfer of electrons and/or protons, are presented in Scheme 1, using the LigandScout 4.4 Expert software platform by InteLigand Softwareentwicklungs- and Consulting GmbH (Austria, Vienna).

**Figure 1:**
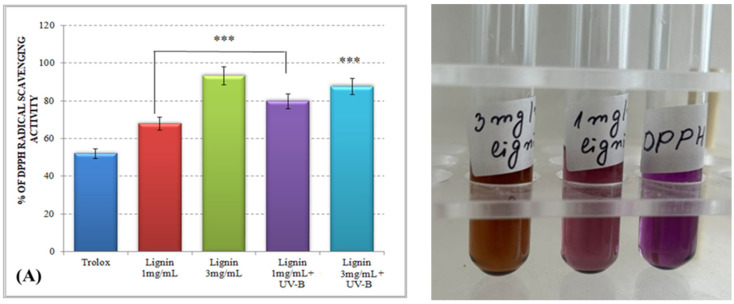

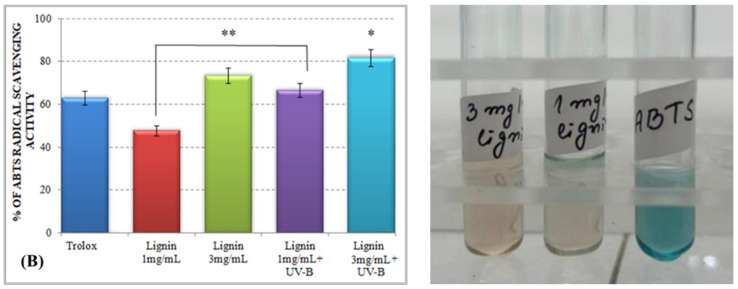
Total antioxidant activity of alkali lignin aqueous solutions, before and after UV-B irradiation, measured by standard methods DPPH radical scavenging activity (**A**) and ABTS radical scavenging activity (**B**). Two levels of significance were considered: * *p* < 0.05 vs. control (water-soluble Trolox) and ** *p* < 0.01 vs. irradiated 1 mg/mL lignin solution. It was not observed the level of statistical significant *** *p* < 0.001.

The results for the total antioxidant activity of water-soluble alkali lignin, obtained by both applied methods, are represented in Figure 1 and displayed that, compared to the control, the antioxidant potential of the heterobiopolymer increased directly with increasing concentrations. The higher total antioxidant activity of lignin, as compared to that of Trolox, could be explained by the possible stabilization of the formed phenoxyl radical, which could be achieved by different types of aromatic ring substituents. For example, *o*-substituents, such as methoxyl groups, stabilize phenoxyl radicals by resonance as well as by hindering them from propagation. Conjugated double bonds can provide additional stabilization of the phenoxyl radicals through extended delocalization. However, a conjugated carbonyl group causes a negative effect on the antioxidant activity of lignin [34]. 

Lignin aqueous solutions with concentrations of 1 mg/mL (0.1%) and 3 mg/mL (0.3%) were subjected to direct UV-B irradiation. The observed results displayed an improvement of the lignin-scavenging capacity towards the stable ABTS radical cation (ABTS^•+^) as well as towards the *p*-radical structure of DPPH (DPPH^•^). Similar results have been reported by Gerbin et al. [35].

**Scheme 2:**
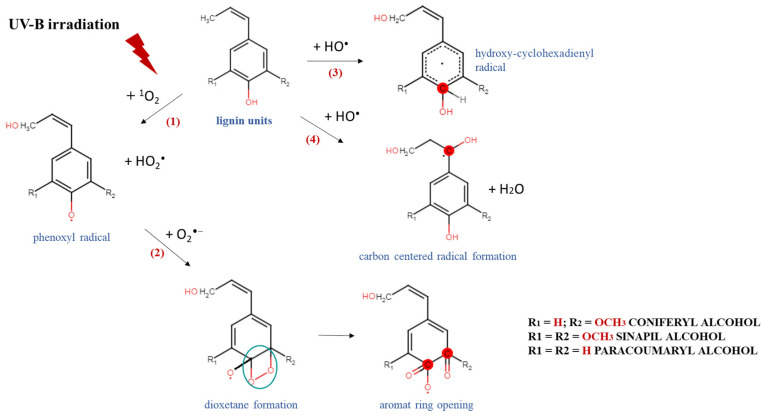
Degradation and/or modification of lignin structure and formation of different radicals after exposure of alkali lignin aqueous solutions to direct UV-B irradiation.

These results could be explained by a possible mechanism of degradation and/or modification of the complex chemical structure of lignin and the generation of different lignin radical formations, depending on the type of reactive oxygen species produced after irradiation [36]. An exposure of the aqueous lignin solutions to UV-B light provides an appropriate environment for the promotion of singlet oxygen formations (^1^O_2_), which are able to react directly with the phenolic groups of lignin molecules, leading to the generation of phenoxyl radicals (Scheme 2, Reaction (1)) [37,38]. Because of its high reactivity within a short timeframe, singlet oxygen undergoes a chemical transformation to initiate a cascade of radical-induced reactions, in which other types of reactive oxygen species, like superoxide (O_2_^−•^) and hydroxyl radicals (OH^•^), are produced [39,40]. The consequent interaction between the formed lignin phenoxyl radicals and superoxide radicals leads to dioxetane formations (Scheme 2, Reaction (2)), which provokes the rearrangement of the lignin aromatic structure and induces an aromatic ring opening [41,42]. On the other hand, due to the highly oxidizing ability of ^•^OH and its electrophilic nature, it could react preferentially by an addition reaction mechanism with the electron-rich aromatic structure of lignin, rather than by outer-sphere electron transfer, which leads to the formation of hydroxy cyclohexadienyl radicals (Scheme 2, Reaction (3)) [43]. Then, a contribution of hydroxy cyclohexadienyl radicals into different type of reactions, such as hydroxylation, demethoxylation, phenolic coupling, and cleavage of a conjugated double bond, could be initiated [36]. The hydroxyl radicals have one more option for interaction with the lignin molecule—hydrogen atom abstraction from a saturated carbon atom from the side chain leading to the production of carbon-centered radical formations and water release (Scheme 2, Reaction (4)) [44]. All abovementioned lignin radicals formed after UV-B irradiation of aqueous alkali lignin solutions possessed a higher scavenging potential against ABTS and DPPH radicals than the nonirradiated samples—a fact that contributed to their more advanced antioxidant activity.

### 2.2. Ferric Ion Reducing Antioxidant Potential of Alkali Lignin Aqueous Solutions

**Figure 2:**
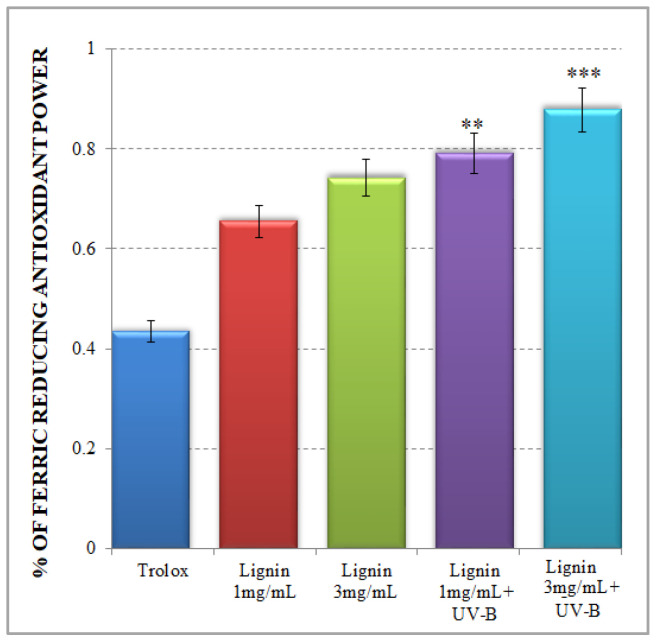
Ferric-ion-reducing antioxidant potential of alkali lignin aqueous solutions. Two levels of significance were considered: ** *p* < 0.01, *** *p* < 0.001; vs. control (water-soluble Trolox).

The FRAP test is a typical non-radical single electron transfer (SET) reaction-based method, which is implemented in acidic pH conditions to facilitate a process of Fe^3+^ reduction to an Fe^2+^ complex in the presence of an antioxidant [33]. In the present study, the observed tendency for an increased ferric-reducing antioxidant potential of alkali lignin aqueous solutions, dependent on the tested concentrations, is presented in Figure 2. Our results confirm the relative high antioxidant activity of lignin, which is associated with its possibility to be involved in the donation of an electron to Fe^3+^ to form an Fe^2+^ structure. There are different studies, which demonstrated the same ferric-ion-reducing ability of lignin and its derivatives because of the possible depolymerization processes of the lignin structure [45,46,47]. However, considerably increased ferric-ion-reducing antioxidant potential of lignin is noticed after UV-B irradiation (Figure 2) especially for the more concentrated sample.

**Scheme 3:**
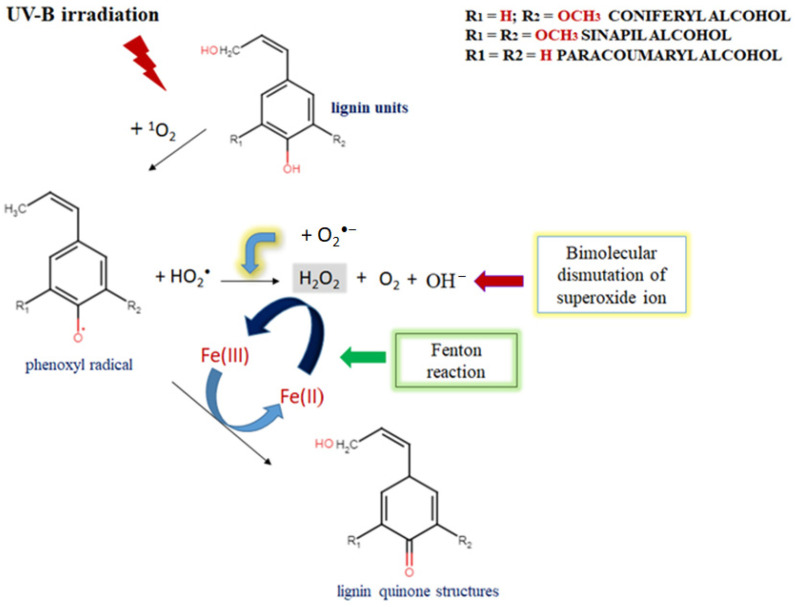
The possible mechanism for upgraded FRAP activity of UV-B-irradiated alkali lignin aqueous solutions and their participation in a Fenton-like reaction.

The improved ferric-ion-reducing potential of lignin aqueous solutions after UV-B irradiation resembles a natural plant-like mechanism of lignin degradation by brown rot fungi, which represents a non-enzymatic oxidation process (Fenton-like reaction) [48,49]. But the critical factor to keep a Fenton system active is making Fe^2+^ available, because its concentration is usually low in natural environments [50,51]. The possible explanation for the upgraded FRAP activity of UV-B-irradiated alkali lignin aqueous solutions, is represented in Scheme 3. As mentioned earlier, UV light initiates singlet oxygen formation, which contributes to phenoxyl radical generation and the production of perhydroxyl radicals as a result of the direct reaction between a lignin molecule and high reactive oxygen species. Additionally, due to the possible effect of a photochemical reaction with water-soluble organic chromophores, such as lignin [52,53,54], superoxide radicals could also be generated [55,56]. As a result, a bimolecular dismutation process occurs, where superoxide radicals are generally used as the main source for hydrogen peroxide formation in water. This process provides the chemical oxidation of Fe^2+^ to Fe^3+^ form and supports the Fenton reaction activity [57,58]. As a consequence, various quinone structures, which are key intermediates in lignin degradation process [59,60], are probably generated and allow the Fenton reaction to continue [61,62].

### 2.3. Effect of Alkali Lignin Solutions on Colon Cancer Cell Viability and Proliferative Activity

**Figure 3:**
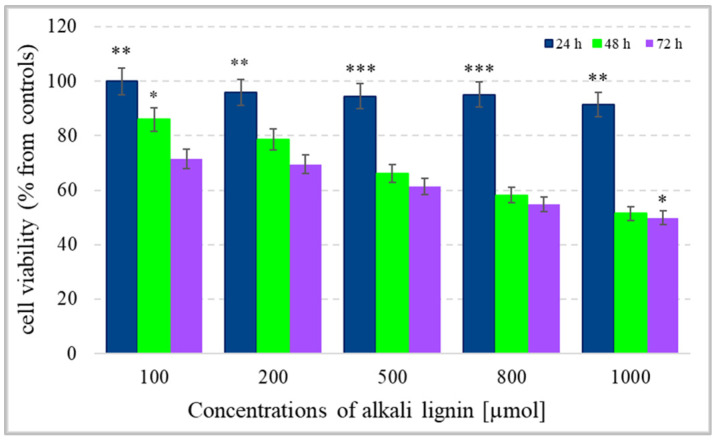
The effect of different concentrations of alkali lignin aqueous solutions on cell viability (metabolite activity) of an epithelium colon cancer cell line, after 24, 48 and 72 h incubation periods. Three levels of significance were considered: * *p* < 0.05, ** *p* < 0.01, *** *p* < 0.001 vs. period of incubation.

**Figure 4:**
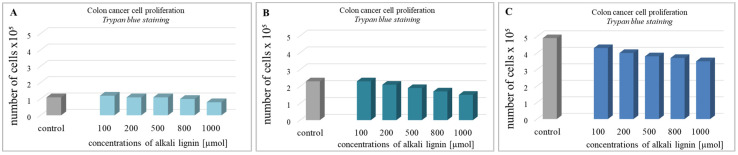
The effect of different concentrations of alkali lignin aqueous solution on the proliferative activity of epithelium colon cancer cell line after 24 (**A**), 48 (**B**) and 72 (**C**) h.

For the determination of colon cancer cell viability (metabolite activity), the standard colorimetric MTT assay method, based on the formation of purple-colored formazan crystals after the reaction between intracellular NAD(P)H-dependent oxidoreductase enzymes and tetrazolium salt, was used [63]. The cells were incubated for 24, 48, and 72 h with water-soluble alkali lignin, applied at five different concentrations and the absorption of dissolved formazan crystals, obtained during each incubation period, was measured. The results in Figure 3 express the reduction of cell viability with increase in alkali lignin concentrations as well as prolonged period of incubation. The fifty percent reduction of cancer cell metabolic activity was observed at 800 µM and 1000 µM concentrations of alkali lignin after 48 and 72 h incubation, respectively. Additionally, the trypan blue staining method was also applied for verification of the effect of alkali lignin on cancer cell growth. Reduction of the proliferative activity was observed at high concentrations, but the effect was not associated with a dramatically decrease in cancer cell growth (Figure 4). However, additional experiments need to clarify the potential toxic effects on normal (non-cancer) cells as well as the induction of hypoxia, which have still been under investigation.

Because of its biodegradable and biocompatible nature, lignin-based chemicals and materials have provoked the attention of the pharmaceutical and healthcare industry. As we described above, the excellent antioxidant activity of lignin compounds provides a basis for their future thorough investigation as a drug-delivery system for various inflammatory diseases, including malignant diseases [64]. In recent years, various publications, which confirm our results and also demonstrate the effectiveness of lignin-based nanoparticles on cancer treatment, have been reported [65,66,67]. Different natural compounds isolated from plant extracts, which possess anti-inflammatory effects, have the potential to inhibit NOX (Nicotinamide adenine dinucleotide phosphate oxidase) activity and to decrease reactive oxygen species (ROS) production [68,69]. There is indisputable evidence about the role of oxidative stress during cancer initiation as well as during malignant progression process [70,71], and various strategies for the elimination of excess ROS levels have been elaborated. The focus of some therapeutic methods have been based on ROS scavenging using antioxidants or drugs which enhance intracellular antioxidant systems [72,73]. Instead, the reduction of oxidative stress through targeting the main ROS cellular sources have been recently reported [74,75]. Various investigations have shown that the activated NOX NADPH oxidases are considered to be initiators of oxidative-induced damage, due to the production of ROS during pathological conditions, including cancer [76]. Therefore, recently, a novel therapeutic strategy, in which blocking the undesirable actions of NOX enzymes has a key role, has been widely discussed [77,78,79]. An experimental study demonstrated that gomisin C, a lignin isolated from the fruits of Schizandra chinensis, reduced respiratory burst in rat peripheral neutrophils by the suppression of NADPH oxidase activity and the inhibition of cytosolic calcium (Ca^2+^) release from an intracellular store [80]. These results suggested that the antioxidant activity of lignin as well as its effect on cellular metabolic activity could be responsible for its possible anticancer potential.

**Scheme 4:**
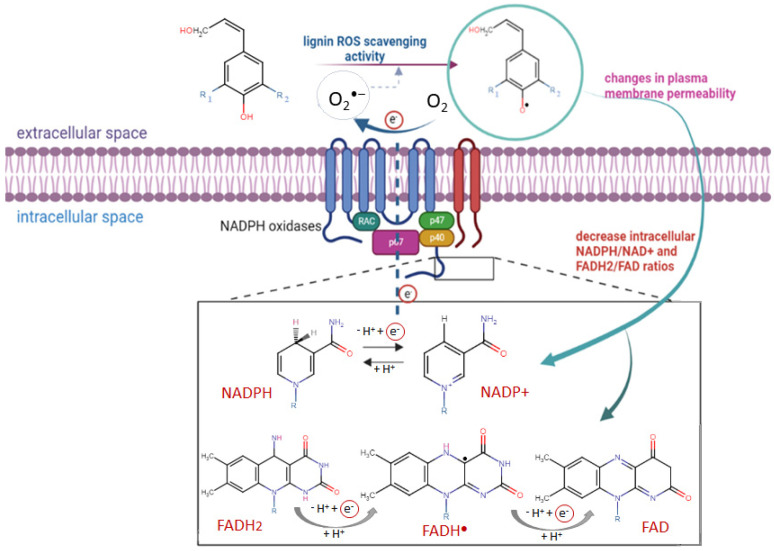
The potential of lignin to inhibit metabolite active cancer cells via interaction between the formed phenoxyl lignin radicals and cofactor forms of plasma membrane NADPH oxidases. The red circle expresses the electron flow through cell membrane.

The probable molecular anticancer mechanism of lignin may include two different aspects (Scheme 4): (i) its direct ROS scavenging activity and/or (ii) a blockage of the enzyme activity of plasma membrane NADPH oxidases. In our study, we demonstrated in vitro the higher antioxidant activity of alkali lignin solutions after direct UV-B irradiation than in a normal environment, through several commonly accepted methods, which have contributed to the explanation of their ROS scavenging activity under induced oxidative stress conditions in cancer cells. We have considered that the repression of the enhanced function of plasma NOX NADPH oxidases by alkali lignin solutions also has the potential to be a key mediator for the decrease in colon cancer cells’ proliferative activity. It has been proved that, being isoforms, plasma NOX family enzymes have a common catalytic core, which is constructed from six transmembrane helices chelating two hemes, binding non-covalently with flavin cofactor (FAD) and the NADPH substrate [77,81]. The function of activated NOX NADPH oxidases is to transport electrons across plasma membranes and to generate large amounts of ROS, when particles, bacteria, and fungi of soluble inflammatory mediators are bound to the specific receptors on the cell surface [82,83]. The possible inhibitor effect of lignin on NOX NADPH oxidases is associated with its high redox potential (up to 1.4 V versus the standard hydrogen electrode) [84,85] and the initiation of chemical reduction reactions of the NADPH substrate or/and FADH_2_ cofactor with phenoxyl lignin radicals, formed due to its ROS scavenging function, thus their oxidized NADP^+^ and FAD forms are produced (Scheme 4). Our assumption is that the reduced cancer cell metabolic activity and inconsistent slight reduction of proliferative activity after treatment with different concentrations of alkali lignin solutions, is probably associated with induced changes in plasma membrane permeability as well as with cell wall extensibility. Similar hypotheses provide challenges, provoking the conduct of extensive research for the clarification of the molecular mechanism(s) of action of lignin and its derivatives and their possible application in biomedicine and cancer treatment.

## 3. Materials and Methods

The LigandScout 4.4 Expert software platform, purchased by the InteLigand Softwareentwicklungs and Consulting GmbH (Vienna, Wien, Austria) is used representing of the images of chemical structures of different lignin structural units as well as the specific functional groups involved into the transfer of electrons and/or protons.

Antioxidant activity against DPPH stable radicals: The hydrophilic analog of vitamin E (Trolox, CAS number: 53188-07-1) as well as the solid substance from lignin alkali (CAS number: 8068-05-1) was purchased from Sigma-Aldrich, Darmstadt, Germany. The assay was performed in accordance with Avelelas et al. [86] with a slight modification, allowing for the testing of the water-soluble alkali lignin solutions and Trolox as a water-soluble control antioxidant standard. Briefly, a stock solution of 2,2-diphenyl-1-picrylhydrazy (DPPH, Sigma-Aldrich, Darmstadt, Germany) in ethanol, at a concentration of 0.1 mM was prepared, for which the initial absorption is approximately 1.0 at 517 nm against ethanol as the blank. Then, 200 µL from sample solutions (water-soluble alkali lignin, 1 mg/mL and 3 mg/mL)/control (Trolox), before and after UV-B irradiation, were added to 3 mL DPPH, incubated in the dark for 30 min and the decrease in absorbance of DPPH at its maximum wavelength was measured spectrophotometrically, using a Hach Lange DR5000 UV/Vis spectrophotometer (Hach Company, Loveland, CO, USA). Triplicate measurements were taken and the radical scavenging activity was calculated using the following formula:

$$DPPH\;\left(\%\right)=\left(1-\frac{Ax}{Ao}\right)\times 100,$$

where *Ax* is the absorbance of the samples/control and *Ao* is the absorbance of the DPPH stock solution.

Antioxidant activity against ABTS standard method: The standard reagent 2,2-azino-bis (3-ethylbenzothiazoline-6-sulphonic acid) (ABTS) was purchased from Sigma-Aldrich, Darmstadt, Germany. The assay was performed in accordance with Re et al. [87], with a slight modification, using Trolox as a standard water-soluble antioxidant. To generate the ABTS radical cation (ABTS^•+^), a mixture of equal volumes of substrate (2,2-azino-bis(3-ethylbenzothiazoline-6-sulphonic acid, 7 mM) and oxidant (potassium persulfate, 2.45 mM) were prepared and kept in the dark for 24 h at 20 °C. Then, the radical cation was diluted with ethanol to prepare a stock solution with initial absorption of 0.7 at 517 nm against ethanol as the blank. Determination of the ABTS-scavenging capacity was achieved after mixing 200 µL of alkali lignin solutions (1 mg/mL and 3 mg/mL), before and after UV-B irradiation with 3.6 mL of prepared ABTS solution. The absorbance of the samples was measured spectrophotometrically, using a Hach Lange DR5000 UV/Vis spectrophotometer (Hach Company, Loveland, CO, USA). Triplicate measurements were taken and the radical scavenging activity was calculated using the following formula:

$$ABTS\;\left(\%\right)=\left(1-\frac{Ix}{Io}\right)\times 100,$$

where *Ix* is the absorbance of the samples/control and *Ao* is the absorbance of the radical cation stock solution.

Ferric-ion-reducing antioxidant potential: The method is described in detail by Benzie and Strain [88] and is based on the electron transfer reaction between the Fe^3+^–TPTZ complex to its oxidized intensive blue-colored form Fe^2+^–TPTZ, in the presence of an antioxidant compound. Freshly prepared FRAP reagent (Sigma-Aldrich, Darmstadt, Germany) contains a mixture of 100 mL of 300 mM acetate buffer, 10 mL of 20 mM FeCl_3_·6H_2_O solution, and 10 mL of 10 mM 2,4,6-tripyridyl-S-triazine (TPTZ), by which the ferric-tripyridyltriazine (Fe^3+^–TPTZ) complex is formed. Briefly, 700 µL of alkali lignin aqueous solution, with concentrations of 1 mg/mL and 3 mg/mL, were added to 2.4 mL of FRAP reagent, incubated for 30 min in the dark and 37 °C and then the absorption was measured at 530 nm using a Hach Lange DR5000 UV/Vis spectrophotometer (Hach Company, Loveland, CO, USA).

UV-B irradiation: The experiment was performed to achieve in vitro a carefully controlled environment for the generation of reactive oxygen intermediates into water solutions of alkali lignin, to estimate their antioxidant potential as well as their redox capacity under induced oxidative stress, using standard methods [36,89]. All water-soluble samples were irradiated at a distance from the light source of 25 to 35 cm, using UVB–vis Transilluminator4000, Starna Cells, Inc, Atascadero, CA, USA, (emitting between 290 nm and 320 nm (peak 309 nm)). To allow UV transparency, to prevent solution evaporation, as well as to keep the samples in a horizontal position, a quartz cover was used. All samples were irradiated over a wide UV-B radiation range (0 to 12 kJ m^−2^) without visible ray, at less than 291–293 K. The UV-B irradiated energy was controlled with an exposure time of 120 min. Dark, fresh air and 46% relative humidity was circulated in the illuminator throughout the irradiation course.

Cells and treatment protocol: The in vitro experiments were performed on a colon cancer epithelial cell line (Colon 26), kindly provided by the Institute of Biophysics and Biomedical Engineering, Bulgarian Academy of Sciences. The standard protocol for the cultivation of adhesive colon cancer cells was used: Dulbecco’s Modified Eagle’s medium (DMEM, Sigma-Aldrich, Darmstadt, Germany), supplemented with a high concentration of glucose (4500 mg/L), 10% heat-inactivated fetal bovine serum (FSB, Sigma-Aldrich, Darmstadt, Germany), and antibiotics (100 U/mol penicillin and 100 µg/mol streptomycin), in a humidified atmosphere, 37 °C and 5% CO_2_. Twenty-four hours before the experiment, the cells were trypsinized, centrifuged (800× *g*/5 min), counted (using the trypan blue method), normalized to 5 × 104 cells per 1 mL and re-suspended in fresh medium without antibiotics in 24-well plates, in order to eliminate their potential effect(s) on the experimental endpoint. The cells were exposed to different low (tolerable) concentrations of alkali lignin water solutions (100 µM, 200 µM, 500 µM, 800 µM, and 1000 µM) to assess their viability and proliferative activity. In the experiments, untreated colon cancer epithelium cells (Colon 26) were used as controls.

Cancer cell viability: The 3-(4,5-dimethylthiazolyl-2)-2,5-diphenyltetrazolium bromide (MTT, Sigma-Aldrich, Germany) assay was used to identify viable cells through measurement of absorption (at 570 nm) of the formed formazan crystals after 3 h incubation in dark with organic dye, using a Zenyth 200rt microplate reader (Tecan Group Ltd., Männedorf, Switzerland). The amount of formazan produced is proportional to the number of live cells in each well. The cell viability of cancer cells after treatment was presented as a percentage of the control cells.

Cancer cell proliferative activity: The metabolite active cells (proliferating cells) were analyzed using the trypan blue (Sigma-Aldrich, Darmstadt, Germany) staining method and an EVE Automated Cell Counter (NanoEntek, Inc., Gyeonggi-do, Korea). Three independent experiments (with two repetitive measurements) were performed for each sample. Non-treated cells were used as controls.

Statistical analysis: All results are expressed as mean ± standard deviation (SD) from three independent experiments with two parallel samples for each experiment (*n* = 6). Comparison between controls and alkali lignin solutions, before and after UV-B irradiation, as well as treated cells, were performed using the Student’s *t*-test. A value of *p* ˂ 0.05 was considered as a significant.

## 4. Conclusions

In conclusion, the specific and selective inhibition of NOX NADPH oxidase isoforms may provide opportunities for the therapeutic treatment of cancer as well as a large number of inflammatory diseases. Our results confirm the potential of lignin and its derivatives to be profoundly studied as a possible redox-sensitive NOX NADPH oxidase inhibitors and/or possible drug nano-carriers. These data once again corroborate the necessity for the elaboration of a novel therapeutic strategy, in which the focus is on the general intracellular ROS sources as the main targets. The growing interest of academia and the pharmaceutical industry towards the application of lignin and its derivatives provides a basis for conducting in-depth studies on their redox-molecular mechanisms in cancer and normal cells. Besides contributing to the design of anticancer therapies, the knowledge gathered in this research could open new gates in the development of food packages or the creation of lignin-based materials with improved moisture barrier properties.

## Data Availability

The data presented in this study are available on request from the corresponding author.
